# Association Between Heavy Metals Exposure and Elevated High-Sensitivity C-Reactive Protein: Mediating Role of Body Mass Index

**DOI:** 10.3390/biom15111491

**Published:** 2025-10-23

**Authors:** Seong-Uk Baek, Jin-Ha Yoon

**Affiliations:** 1 Graduate School, Yonsei University College of Medicine, Seoul 03722, Republic of Korea; 2 Gwansan Public Health Unit, Jangheung County Public Health Center, Jangheung 59014, Republic of Korea; 3 Department of Preventive Medicine and Public Health, Yonsei University College of Medicine, Seoul 03722, Republic of Korea

**Keywords:** inflammation, systemic inflammation, heavy metal, adiposity

## Abstract

Heavy metal exposure is linked to obesity and systemic inflammation. This study explored the mediating role of body mass index (BMI) in the association of heavy metal exposure with high-sensitivity C-reactive protein (hs-CRP). Blood levels of mercury (Hg), cadmium (Cd), and lead (Pb) were assessed in a nationwide sample of 4521 adults. Linear regressions were employed to examine associations among blood heavy metal levels, BMI, and hs-CRP levels. Mediation analyses were conducted to estimate the indirect effect of exposure to each heavy metal on the elevation of hs-CRP through an increase in BMI. The median (Q1; Q3) values for blood levels of heavy metals were 3.15 (2.10; 4.84) for Hg (μg/L), 0.95 (0.63; 1.38) for Cd (μg/L), and 1.67 (1.28; 2.21) for Pb (μg/dL). Blood Hg level was associated with both BMI (adjusted *β*: 0.73; 95% CI [confidence interval]: 0.51; 0.96) and a log-transformed hs-CRP level (adjusted *β*: 0.07; 95% CI: 0.02; 0.13). Blood Cd and Pb levels showed no clear associations with BMI and hs-CRP. The indirect effect of Hg exposure on hs-CRP via BMI was 0.069 (95% CI: 0.037; 0.102), and that of the direct effect was 0.003 (95% CI: −0.001; 0.007), with BMI accounting for 95.7% (95% CI: 88.6%, 102.0%) of the total association between Hg levels and hs-CRP levels. Hg exposure was linked to increased hs-CRP levels, with elevated BMI explaining most of this association. This research offers insights into the mechanisms through which Hg contributes to systemic inflammation, underscoring the potential role of BMI as a key mediator.

## 1. Introduction

Heavy metals, such as mercury (Hg), cadmium (Cd), and lead (Pb), are environmental toxic substances that have garnered considerable scholarly and social interest in recent years due to their adverse health effects and ecological impacts [1,2,3,4]. Over the past few decades, regulatory measures in South Korea have gradually decreased environmental exposure to heavy metals. However, research indicates that serum levels of Hg, Cd, and Pb among the Korean population are higher compared to populations in the United States (US), Canada, or Germany [5,6]. Heavy metal exposure is associated with poor health outcomes, including cardiovascular disease [7], metabolic disorders [8], and cancers [9]. The International Agency for Research on Cancer (IARC) classified Cd exposure as having sufficient evidence for lung cancer incidence (Group 1) [10]. Meanwhile, Pb exposure was classified as having limited evidence of its carcinogenic effect on stomach cancer (Group 2A) [11], and Hg exposure was classified as “not classifiable” as to its carcinogenicity to humans [12].

While complex biological pathways may link the relationship between exposure to heavy metals and the onset of adverse health outcomes, oxidative stress and inflammatory responses are considered key factors in this process [13,14]. Blood level of high-sensitivity C-reactive protein (hs-CRP) is not only used to identify acute inflammation, such as that caused by infections, but it is also a widely used marker for low-grade systemic inflammation [15]. For instance, urinary level of Cd was positively associated with CRP level in US adults [13]. Additionally, a recent study showed that exposure to a heavy metal mixture, which encompasses Hg, Cd, and Pb, was positively associated with CRP levels [16].

Obesity is often accompanied by systemic inflammation. The accumulation of free fatty acids and the hypertrophy of adipose tissue can induce an inflammatory response in adipose tissue, leading to the release of interleukin-6 (IL-6), which, in turn, stimulates hepatic synthesis of C-reactive protein [17]. Systematic analysis confirmed the positive association between body mass index (BMI) and CRP [18]. Moreover, studies based on randomized controlled trials and Mendelian randomization have consistently confirmed the causal effects of BMI on CRP levels [19,20].

Recently, academic interest in the impact of heavy metal exposure on metabolic dysfunction has been growing. Heavy metals, such as Hg, cadmium Cd, and lead Pb, have been classified as metalloestrogens that exert endocrine-disrupting effects on hormone receptors [21]. Research indicates that heavy metals provoke metabolic disruption in adipose tissue, which is an organ essential for maintaining energy homeostasis, thereby establishing a potential link to subsequent changes in body weight [22,23]. Mounting evidence suggests that exposure to heavy metals may contribute to the disruption of adipocytes and induce obesity [22,24,25,26]. For instance, previous research showed that exposure to heavy metal mixture was linked to increases in BMI and increased risk of obesity [27,28,29,30], while these associations varied substantially depending on the type of heavy metal [31,32]. Notably, exposure to Hg was consistently linked to a higher risk of obesity and increased BMI in recent meta-analytic reviews [33,34]. In addition to this epidemiological evidence, experimental studies have provided mechanistic insights into how Hg exposure can lead to obesity. Previous studies suggest that exposure to Hg can induce white adipose tissue dysfunction [26] or upregulate adipogenic genes [35], which can contribute to obesity.

**Figure 1:**
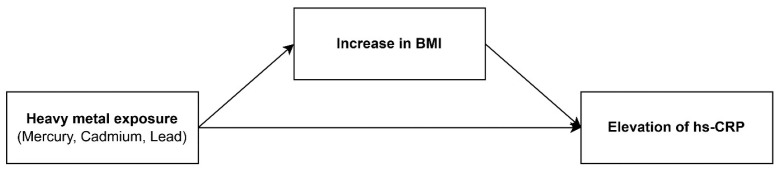
The mediating role of body mass index (BMI) in the association between heavy metals.

Based on existing evidence that heavy metal exposure is associated with increases in BMI and CRP levels, and considering that adiposity (reflected by increased BMI) can cause elevations in hs-CRP, BMI may serve as a mediator in the relationship between heavy metal exposure and hs-CRP levels. However, studies addressing this topic remain scarce in the literature. Figure 1 presents a conceptual framework that outlines the potential associations among heavy metal exposure, BMI, and hs-CRP. Accordingly, the objective of this study was to examine the mediating role of BMI in the association between heavy-metal exposure and hs-CRP levels, using data from a nationwide sample of Korean adults.

## 2. Materials and Methods

### 2.1. Study Sample

This study utilized the study sample from the Korea National Health and Nutrition Examination Survey (KNHANES) [36]. The KNHANES is an annually repeated cross-sectional survey with the objective of gathering information on the health of people living in Korea. The 2016 and 2017 datasets of the KNHANES, in which blood heavy metals (Hg, Cd, Pb) and hs-CRP had been measured simultaneously, were analyzed. The KNHANES employed a multi-staged clustered probability sampling design to include a nationwide sample of the Korean population. The sampling procedure involved selecting 192 regions covering Korea, and households in each region were systematically sampled for participation in the study. The household-based health examinations, including surveys and blood tests, were conducted by trained health professionals. Of the 4831 adult participants whose blood heavy metal levels were measured, 310 participants with missing values were excluded, leaving a total of 4521 Korean adults included in the analysis for this study. The KNHANES datasets are accessible at https://knhanes.kdca.go.kr, accessed on 1 July 2025. This study was granted IRB exemption by Yonsei University Healthy System (No. 4-2024-1135).

### 2.2. Variables

Blood samples were obtained following an eight-hour fasting period using venipuncture. The collected samples were stored at 2–6 °C and subsequently transported to the central laboratory for analysis. A 3 mL aliquot of blood was drawn into Trace Element EDTA tubes containing sodium heparin (BD Vacutainer K2-EDTA tubes; Becton Dickinson, Franklin Lakes, NJ, USA). Blood Pb and Cd concentrations were determined via graphite furnace atomic absorption spectrometry (GFAAS) equipped with Zeeman background correction (AAnalyst 600; PerkinElmer, Turku, Finland). For sample pretreatment, 0.1 mL aliquots of blood were diluted twentyfold with 0.2 (*w*/*v*)% (NH_4_)_2_HPO_4_ containing 0.1 (*v*/*v*)% Triton X-100, and 15 μL of the diluted sample was introduced into the graphite furnace [37]. Blood mercury (Hg) levels were quantified using the gold amalgamation technique combined with thermal decomposition atomic absorption spectrophotometry (DMA-80; Milestone, Bergamo, Italy). The instrumental parameters were optimized for Pb (wavelength: 283 nm, slit width: 0.7 nm, lamp current: 440 mA), Cd (wavelength: 228.8 nm EDL lamp, slit width: 0.7 nm, lamp current: 220 mA), and Hg (wavelength: 253.7 nm). Whole blood metals control (Bio-Rad, Hercules, CA, USA) and blood metals control from the German External Quality Assessment Scheme (G-EQUAS, Erlangen, Germany) were used for internal quality assurance and control, respectively. The inter-assay coefficients of variation were 0.95–4.82% for Pb, 2.65–6.50% for Cd, and 1.59–4.86% for Hg. Calibration was accepted when the mean of three measurements achieved an R^2^ ≥ 0.995. External quality assessments based on the CDC External Quality Assessment (CDC-LAMP) program were conducted annually. According to the quality control reports from the 2016 and 2017 KNHANES, these procedures ensured the precision and accuracy of the quantification process [38]. The limits of detection (LOD) were 0.158 μg/L for Hg, 0.056 μg/L for Cd, and 0.12 μg/dL for Pb, while the limits of quantification (LOQ) were 0.527 μg/L, 0.187 μg/L, and 0.40 μg/dL, respectively. None of the samples were below the LOD. A total of 0.31% for Hg, 0.89% for Cd, and 0.1% for Pb had concentrations between the LOD and LOQ. As this study focused on epidemiological data analysis rather than individual exposure assessment, these estimated values were treated as continuous variables and included in the analyses rather than being discarded [39].

BMI was calculated using the standard formula: weight (kg)/height^2^ (m^2^). Based on a standardized protocol, body weight and height were assessed by a GL-6000-20 (G-Tech, Uijeongbu-si, Republic of Korea) and a Seca 274 stadiometer (SECA, Hamburg, Germany), respectively. Weight and height measurements were recorded to the nearest 0.1 kg and 0.1 cm, respectively.

Blood concentrations of high-sensitivity C-reactive protein (hs-CRP, mg/L) were assessed using immunoturbidimetry with a Cobas analyzer (Roche Diagnostics, Mannheim, Germany). The LOD was 0.15 mg/L, and the values below the LOD were replaced with half of the LOD value.

### 2.3. Statistical Analysis

#### 2.3.1. Preliminary Analysis

First, this study explored how heavy metal levels were associated with participants’ BMI and hs-CRP levels. Heavy metal concentrations and hs-CRP levels were natural log-transformed to approximate a normal distribution, consistent with previous studies [27,28,29,30]. The associations of blood heavy metal levels with BMI and log-hs-CRP levels were explored using linear regression models. Multivariate models were adjusted for the following sociodemographic features: sex (male, female), residential area (urban, rural), age (continuous scale), education level (elementary school or below, middle school, high school, college or above), income level (lowest, low, medium, high, highest), economic activity (active, inactive), marital status (married, unmarried or others), current smoking status (yes, no), physical activity (no, yes), current alcohol use (yes, no), and chronic condition (yes, no). The quintile values of the monthly household income for each year (2016 or 2017) were used to categorize income levels. The current status of smoking and alcohol use was self-reported. Physical activity was categorized into no or yes, whether respondents regularly engaged in 150 min or more of moderate-to-vigorous aerobic physical activity, with a Global Physical Activity Questionnaire [40,41]. Chronic condition was grouped into yes or no, based on the presence of at least one of the following diseases: cerebrovascular disease, cardiovascular disease, tuberculosis, asthma, diabetes, hepatic B or C virus infection, and chronic kidney disease. To address potential multicollinearity that may arise from including multiple blood heavy metal concentrations in the regression model, this study confirmed that Pearson’s correlations between the log-transformed concentrations of blood heavy metals were below 0.5. Additionally, for all multivariate models, the absence of multicollinearity was confirmed by ensuring that the variance inflation factor remained below the threshold of 4 [42].

#### 2.3.2. Mediation Analysis

After observing a positive association of each heavy metal with BMI and hs-CRP levels, this study carried out a mediation analysis with the R package “mediation.” The methodology of mediation analysis is described in the earlier literature [43,44]. This involved the decomposition of total effect (TE) into direct effect (average direct effect, ADE) and indirect effect (average causal mediation effect, ACME) (TE = ADE + ACME). The 95% confidence intervals (CIs) were determined based on quasi-Bayesian approximation with 1000 Monte Carlo simulations. Survey weights were incorporated into statistical analyses using the R package “survey”. Additionally, this study conducted a sensitivity analysis based on multiple imputations to handle missing values. Twenty datasets with complete information were generated using the multiple imputations by chained equations (MICE) method; analyses were performed on each dataset, and then the estimates were combined. The imputation was performed using the R package “mice” [45]. All analyses were executed using the R software, version 4.4.1.

## 3. Results

The sample consisted of 1989 men (44.0%) and 2532 women (56.0%) with a mean age of 49.9 (standard deviation: 16.4; Table 1). Table S1 shows the features of participants included and excluded from analyses. Compared with the included sample, those excluded were more likely to be men, older, with lower education and income levels. While blood Cd levels were higher among those in the excluded sample, no clear differences were confirmed in the blood concentrations of Hg, Pb, hs-CRP, and BMI.

Table 2 shows the descriptive statistics of blood heavy metal and hs-CRP concentrations and BMI of the study sample. The medians (Q1, Q3) for blood Hg (μg/L), Cd (μg/L), and Pb (μg/dL) levels, BMI (kg/m^2^), and blood hs-CRP level (mg/L) were 3.15 (2.10, 4.84), 0.95 (0.63, 1.38), 1.67 (1.28, 2.21), 23.71 (21.51, 26.10), and 0.60 (0.38, 1.14), respectively. The geometric means for blood Hg (μg/L), Cd (μg/L), and Pb (μg/dL) levels were 3.22, 0.91, and 1.68, respectively. Positive correlations were observed among the sample’s heavy metal concentrations, BMI, and hs-CRP levels.

Table 3 shows the association of heavy metal concentrations with BMI and hs-CRP levels. In the multivariate models, a natural-log increase in blood Hg level was associated with an increase in BMI (*β*: 0.73; 95% CI: 0.51, 0.96), while Cd and Pb levels showed no clear associations with BMI. Similarly, in the multivariate models, a natural-log increase in blood Hg level was positively associated with a hs-CRP level (*β*: 0.07; 95% CI: 0.02, 0.13). Table S2 shows that BMI was also positively related to hs-CRP levels.

**Figure 2:**
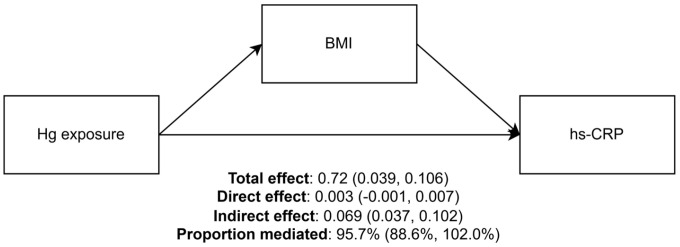
The mediating role of body mass index in the association between blood mercury level and high-sensitivity C-reactive protein (hs-CRP). **Notes**: Blood mercury and hs-CRP levels were natural log-transformed. Models were controlled for sex, region, age, education, income, marital status, economic activity, smoking status, physical activity, alcohol use, chronic condition, and log-transformed concentrations of blood cadmium and lead.

Figure 2 shows the results of the mediation analysis. Considering that blood Hg was the only variable exhibiting positive associations with both BMI and hs-CRP levels, mediation analysis was conducted with Hg treated as the exposure variable, while Cd and Pb levels and sociodemographic variables were considered covariates. The estimates (95% CI) were 0.072 (0.039, 0.106) for TE, 0.003 (−0.001, 0.007) for ADE, and 0.069 (0.037, 0.102) for ACME. This indicates that a natural logarithm increase in blood Hg was associated with a 0.072 increase in outcome, of which 0.069 was attributable to the increase in BMI. The proportion mediated by BMI was estimated to be 95.7% (95% CI: 88.6%, 102.0%).

## 4. Discussion

The results indicate that blood Hg levels were positively associated with both BMI and hs-CRP levels. Moreover, increases in BMI explained approximately 96% of the association of mercury exposure with elevated hs-CRP levels. This study provided insights into the relationship between heavy metal exposure and inflammatory response, highlighting the role of BMI in this relationship.

The results indicated a positive correlation between blood Hg levels and BMI, which is consistent with previous studies. For instance, a systemic meta-analysis demonstrated that mercury exposure is linked to higher odds of obesity and abdominal obesity [33]. Another meta-analysis explored the associations of various heavy metals with obesity and found that only Hg exposure exhibited a positive association with obesity [34]. These findings also corroborate those of prior studies showing that exposure to Hg is positively correlated with the indicators of visceral adiposity [27,46]. Additionally, the findings align with prior research showing that Hg exposure is linked to increased inflammatory marker levels. For instance, blood Hg levels were positively correlated with hs-CRP [47], IL-6 and interferon-γ [48], and inflammatory profiles of white blood cells [49]. A study from the US showed that, while a mixture of heavy metals is associated with CRP levels, exposure to Hg may contribute the most to this positive association [16].

Although the existing literature does not elucidate the precise mechanisms by which Hg exposure leads to increases in BMI, the underlying processes are likely complex [22,24,25,26]. For instance, a previous study showed that prolonged exposure to Hg was linked to an impaired white adipose tissue function [26]. Rats chronically exposed to Hg exhibited a reduction in the size of white adipose tissue, along with increases in plasma triglycerides and total cholesterol, and disrupted mRNA expression of adipokines, suggesting an obesogenic effect of Hg exposure [26]. Additionally, chronic exposure to MeHg was linked to upregulation of adipogenic genes and accumulation of triglyceride in the adipocytes [35]. Consequently, Hg-induced adipogenesis can lead to obesity and increases in BMI, which could, in turn, result in the release of inflammatory cytokines and an increase in hs-CRP levels.

Previous studies have reported that South Korea has higher levels of heavy metal exposure than North America or European regions [5,6]. In this study, the geometric mean blood Pb level (1.67 μg/dL) was higher than that observed in the U.S. population (0.82 μg/dL) during a similar period (NHANES 2015–2016) [50]. In addition, the median blood Cd and Hg levels were also higher than those reported in NHANES 2015–2016 (0.23 μg/L and 0.60 μg/L, respectively) [51]. Such elevated heavy metal levels in Korea have been attributed to factors such as dietary practices and environmental exposures [52,53]. Therefore, these findings suggest the need for efforts to reduce heavy metal exposure in Korea.

This study has limitations. First, the true causal effects that heavy metals exert on BMI and hs-CRP could not be elucidated, as the current analyses were based on a cross-sectional study design. The reciprocal relationship between heavy metal exposure and body weight should be considered. For instance, individuals with obesity may have increased heavy metal deposition, and the endocrine-disrupting potential of these metals may further elevate the risk of weight gain. Therefore, in the future, longitudinal research should explore temporal associations among heavy metal exposure, BMI, and hs-CRP. Second, the measurements for heavy metals may not accurately capture the long-term exposure levels, because blood samples were collected only once for each individual. Given the gradual nature of the adipogenesis process in the human body, future research should employ methods, such as repeated measurements, that more accurately reflect chronic exposure levels. Third, owing to the lack of information, this study could not address factors such as the use of anti-inflammatory drugs (e.g., steroids) or genetic predisposition, which can substantially influence both BMI and hs-CRP levels of participants. Fourth, despite the widespread use of hs-CRP as a biomarker of systemic inflammation, the findings are constrained by the limited assessment of inflammatory markers. To obtain a more complete picture, future studies should employ a more comprehensive panel of inflammatory markers, including TNF-α, IL-1, and IL-6, to better understand the relationship between heavy metal exposure and inflammation. Fifth, this study was limited to only three heavy metals because the KNHANES measured only the concentrations of Hg, Cd, and Pb for participants, and did not include a broader range of heavy metals. Therefore, future research should aim to incorporate a wider variety of heavy metals. Sixth, it has been suggested that BMI has limitations in fully reflecting visceral adipose tissue or total body fat mass [54]. Future in-depth studies utilizing more precise body composition measurement tools are needed to validate the study’s findings. Seventh, the study participants were limited to the Korean population. Therefore, the findings may not be necessarily applicable to populations of other ethnicities or those residing in different geographical areas.

Despite its limitations, this study has the following strengths: First, the analyses were based on a sample that is representative of the Korean population. This may enhance the generalizability of the findings. Second, this study represents one of the first attempts to investigate the mediating role of BMI in the association of heavy metal exposure with hs-CRP, which may enhance the understanding of how heavy metal exposure induces inflammatory conditions and, ultimately, leads to negative health consequences, such as metabolic disorders and cardiovascular diseases.

## 5. Conclusions

This cross-sectional mediation analysis revealed that among the heavy metals Pb, Cd, and Hg, Hg exposure was associated with higher hs-CRP levels in Korean adults, with BMI mediating this association. Although this study was limited to a Korean population and relied on a cross-sectional analysis, which precludes the establishment of true temporal or causal relationships, future studies in other populations and with longitudinal designs are warranted to confirm these findings and elucidate the endocrine-disrupting potential of these heavy metals.

## Figures and Tables

**Table 1:** Study sample characteristics.

N (%)	
N	4521 (100%)
Sex	
Male	1989 (44.0%)
Female	2532 (56.0%)
Region	
Urban	3692 (81.7%)
Rural	829 (18.3%)
Age	
Mean (SD)	49.9 (16.4)
Education level	
Elementary school or below	846 (18.7%)
Middle school	454 (10.0%)
High school	1489 (32.9%)
College or above	1732 (38.3%)
Income	
Lowest	788 (17.4%)
Low	861 (19.0%)
Medium	919 (20.3%)
High	934 (20.7%)
Highest	1019 (22.5%)
Economic activity	
Active	2822 (62.4%)
Inactive	1699 (37.6%)
Marital status	
Married	3130 (69.2%)
Unmarried or others	1391 (30.8%)
Smoking status	
Yes	866 (19.2%)
No	3655 (80.8%)
Physical activity	
Yes	1995 (44.1%)
No	2526 (55.9%)
Alcohol use	
Yes	3017 (66.7%)
No	1504 (33.3%)

**Table 2:** Spearman’s correlations between serum heavy metal concentrations, BMI, and hs-CRP levels.

	Median (Q1, Q3)	Min, Max	Hg	Cd	Pb	BMI	hs-CRP
**Hg** (μg/L)	3.15 (2.10, 4.84)	0.29, 42.80	1				
**Cd** (μg/L)	0.95 (0.63, 1.38)	0.10, 6.62	0.09	1			
**Pb** (μg/dL)	1.67 (1.28, 2.21)	0.20, 20.16	0.28	0.30	1		
**BMI** (kg/m^2^)	23.71 (21.51, 26.10)	15.20, 43.56	0.17	0.06	0.12	1	
**hs-CRP** (mg/L)	0.60 (0.38, 1.14)	0.07, 19.99	0.08	0.08	0.11	0.38	1

All coefficients were significant at the level of *p* < 0.001; Hg, mercury; Cd, cadmium; Pb, lead; SD, standard deviation; BMI, body mass index; hs-CRP, high-sensitivity C-reactive protein.

**Table 3:** Association among heavy metal concentrations, body mass index, and log-transformed high-sensitivity C-reactive protein.

		Dependent Variables							
		BMI				hs-CRP ^a^			
		Univariate Model		Multivariate Model		Univariate Model		Multivariate Model	
Exposure	Mean (SD)	*β* (95% CI)	*p*	*β* (95% CI)	*p*	*β* (95% CI)	*p*	*β* (95% CI)	*p*
**Log-Hg ^a^**	1.17 (0.64)	0.93 (0.75, 1.12)	<0.001	0.73 (0.51, 0.96)	<0.001	0.11 (0.06, 0.16)	<0.001	0.07 (0.02, 0.13)	0.012
**Log-Cd ^a^**	−0.09 (0.60)	0.26 (0.04, 0.48)	0.021	0.20 (−0.10, 0.50)	0.196	0.09 (0.04, 0.14)	0.001	−0.00 (−0.07, 0.06)	0.880
**Log-Pb ^a^**	0.52 (0.42)	0.94 (0.63, 1.26)	<0.001	−0.06 (−0.41, 0.28)	0.727	0.20 (0.13, 0.28)	<0.001	−0.01 (−0.09, 0.07)	0.809

^a^ natural log-transformed; Hg, mercury; Cd, cadmium; Pb, lead; CI, confidence interval; Multivariate model: sex + region + age + education + income + marital status + economic activity + smoking status + physical activity + alcohol use + chronic condition + log-Hg levels + log-Cd levels + log-Pb levels.

## Data Availability

The data are accessible at https://knhanes.kdca.go.kr/, accessed on 1 July 2025.

## Supplementary Materials

The following supporting information can be downloaded at https://www.mdpi.com/article/10.3390/biom15111491/s1, Table S1: Characteristics of participants included and excluded from analyses; Table S2: Association between body mass index and hs-CRP; Table S3: The mediating role of body mass index in the association between blood mercury level and high-sensitivity C-reactive protein (hs-CRP).
