# Supplementary material for: Association Between Heavy Metals Exposure and Elevated High-Sensitivity C-Reactive Protein: Mediating Role of Body Mass Index
Source: Biomolecules. 2025 Oct 23;15(11):1491. doi: 10.3390/biom15111491 (PMC12650004; doi:10.3390/biom15111491)
Supplement: Supplementary file 1 [file biomolecules-15-01491-s001.zip › biomolecules-3913520-supplementary.pdf]

Table S1 Characteristics of participants included and excluded from analyses

	Excluded sample	Included sample	*P*[a]
N	310 (6.4%)	4,521 (100%)	
Sex			0.011
Male	160 (51.6%)	1989 (44.0%)	
Female	150 (48.4%)	2532 (56.0%)	
Region			
Urban	246 (79.4%)	3692 (81.7%)	
Rural	64 (20.6%)	829 (18.3%)	
Age			0.001
Mean (SD)	53.0 (16.3)	49.9 (16.4)	
Education level			< 0.001
Elementary school or below	29 (46.0%)	846 (18.7%)	
Middle school	7 (11.1%)	454 (10.0%)	
High school	15 (23.8%)	1489 (32.9%)	
College or above	12 (19.0%)	1732 (38.3%)	
Missing	247		
Income			0.061
Lowest	71 (24.1%)	788 (17.4%)	
Low	54 (18.3%)	861 (19.0%)	
Medium	51 (17.3%)	919 (20.3%)	
High	60 (20.3%)	934 (20.7%)	
Highest	59 (20.0%)	1019 (22.5%)	
Missing	15		
Economic activity			0.011
Active	30 (46.2%)	2822 (62.4%)	
Inactive	35 (53.8%)	1699 (37.6%)	
Missing	245		
Marital status			0.063
Married	230 (74.4%)	3130 (69.2%)	
Unmarried or others	79 (25.6%)	1391 (30.8%)	
Missing	1		
Smoking status			0.082
Yes	57 (23.9%)	866 (19.2%)	
No	181 (76.1%)	3655 (80.8%)	
Missing	72		
Physical activity			0.179
Yes	18 (34.0%)	1995 (44.1%)	
No	35 (66.0%)	2526 (55.9%)	
Missing	257		
Alcohol use			0.375
Yes	168 (69.7%)	3017 (66.7%)	
No	73 (30.3%)	1504 (33.3%)	
Missing	69		
Hg (μg/L)			0.178
Median (Q1, Q3)	3.3 (2.2, 5.4)	3.2 (2.1, 4.8)	
Cd (μg/L)			0.017
Median (Q1, Q3)	1.0 (0.7, 1.4)	1.0 (0.6, 1.4)	
Pb (μg/L)			0.169
Median (Q1, Q3)	1.7 (1.3, 2.4)	1.7 (1.3, 2.2)	
BMI (kg/m^2)			0.324
Mean (SD)			
Missing	8		
hs-CRP (mg/L)			0.716
Median (Q1, Q3)	0.6 (0.4, 1.2)	0.6 (0.4, 1.1)	
Missing	25		

[a]Chi-squared test for categorical variables, Wilcoxon rank-sum test for blood concentrations for heavy metals and hs-CRP, and t-test for age and BMI

Table S2 Association between body mass index and hs-CRP

	β (95% CI)	P value
**BMI**	0.09 (0.008, 0.010)	< 0.001

Models were controlled for sex, region, age, education, income, marital status, economic activity, smoking status, physical activity, alcohol use, chronic condition, and log-transformed concentrations of heavy metals

Table S3 The mediating role of body mass index in the association between blood mercury level and high-sensitivity C-reactive protein (hs-CRP). Sensitivity analysis based on imputed datasets.

	β (95% CI)
**TE**	0.065 (0.036, 0.095)
**ADE**	-0.006 (-0.010, -0.001)
**ACME**	0.071 (0.042, 0.100)
**PM (95% CI)**	108.4 % (98.5%, 118.4%)

TE, total effect; ADE, average direct effect; ACME, average causal mediation effect; PM, proportion mediated; CI, confidence interval

Blood mercury and hs-CRP levels were natural log-transformed.

Models were controlled for sex, region, age, education, income, marital status, economic activity, smoking status, physical activity, alcohol use, chronic condition, and log-transformed concentrations of blood cadmium and lead.
